# The characteristics of invasive cardiac lipoma: case report and literature review

**DOI:** 10.3389/fcvm.2023.1195582

**Published:** 2023-07-10

**Authors:** Xiliang Zhu, Zhaoyun Cheng, Sheng Wang, Xianjie Chen, Guoqing Lu, Xiaoyang Li

**Affiliations:** Department of Cardiovascular Surgery, Henan Province People's Hospital, Zhengzhou University, Zhengzhou, People's Republic of China

**Keywords:** cardiac, cardiac tumor, cardiac lipoma, invasive cardiac lipoma, cardiac tumor diagnosis

## Abstract

Invasive cardiac lipoma is a rare type of primary cardiac tumor that is composed of adipose tissue but infiltrating the adjacent structures. It is a benign tumor that can cause significant morbidity and mortality due to its size and location within the heart. We describe a giant invasive intracardiac lipoma across atrial wall extending to the ascending aorta and the superior vena cava. This review will provide an overview of invasive cardiac lipoma, including its clinical presentation, diagnosis, and management.

## Introduction

Invasive cardiac lipoma is a rare, benign tumor that originates from adipose tissue and can develop within the heart. Although benign, these tumors can pose significant clinical challenges due to their infiltration to adjacent structure causing obstruction, arrhythmias, and hemodynamic instability (1, 2). The incidence of cardiac lipoma is 2.4% of all primary cardiac tumors from the report of Japanese Circulation Society, and the invasive cardiac lipoma is rarer (3). However, due to its potential for adverse outcomes, timely diagnosis and management are critical for optimal patient outcomes.

Despite its rarity, invasive cardiac lipoma remains an important clinical entity that requires prompt recognition and management (1). We report a case of typical invasive cardiac lipoma and provide an overview of the epidemiology, clinical presentation, diagnostic modalities, and management of invasive cardiac lipoma, with a focus on recent advances in surgical techniques and outcomes. By enhancing our understanding of this condition, we can improve patient outcomes and optimize management strategies for patients with invasive cardiac lipoma.

## Case report

**Figure 1:**
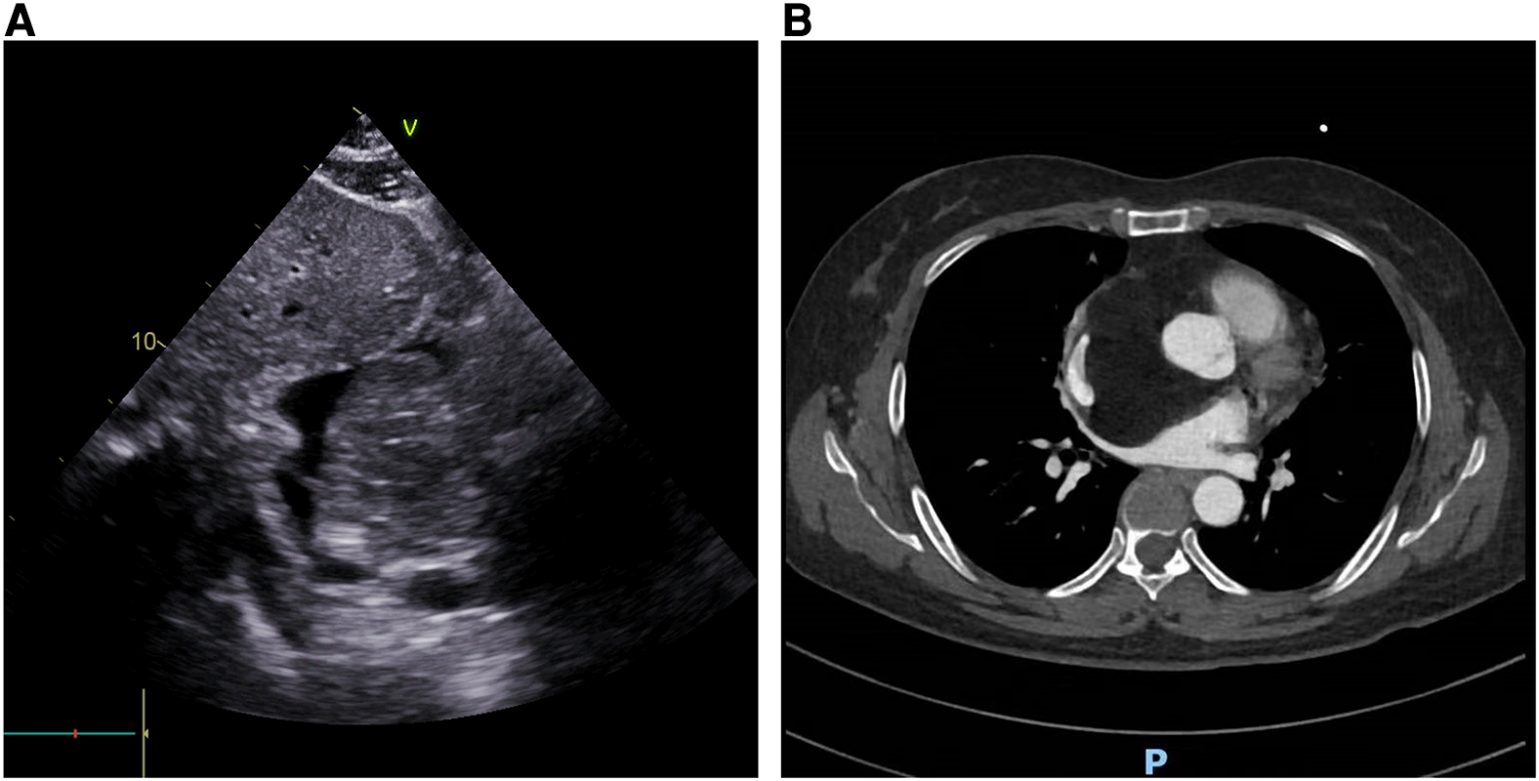
The imaging feature of the cardiac mass. (**A**) Echocardiography revealed a large mass attached to the interatrial septum; (**B**) CT revealed a low-density mass within the atrium, which compressed the superior vena cava and ascending aorta.

**Figure 2:**
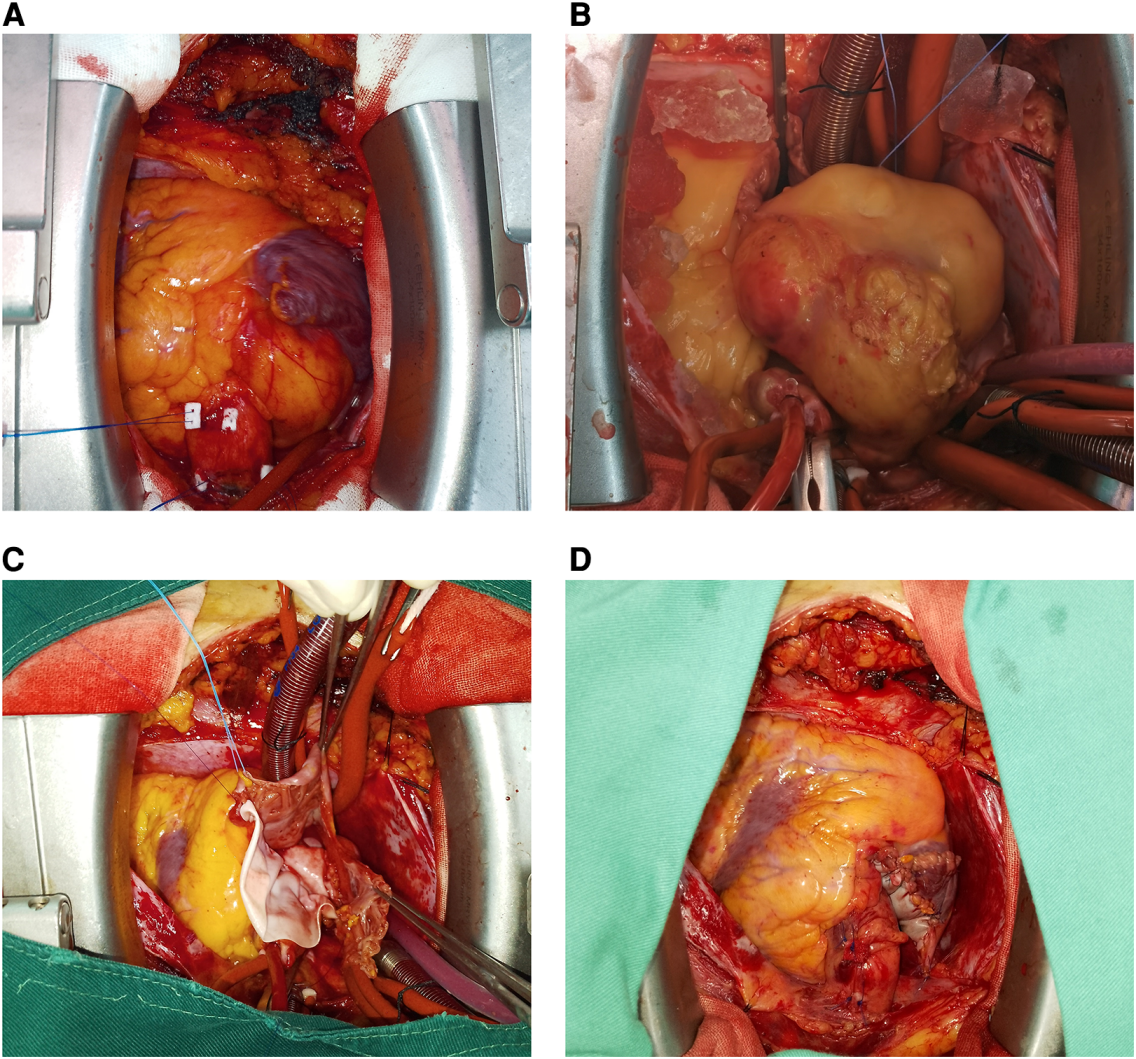
The surgical view of the mass. (**A**) A large yellow mass covering the superior vena cava and the root of the ascending aorta; (**B**) the mass was dissected by suspending it via surgical sutures; (**C**) the mass was found to originate from the interatrial septum after exposing the right atrium; (**D**) the right atrium was sutured.

**Figure 3:**
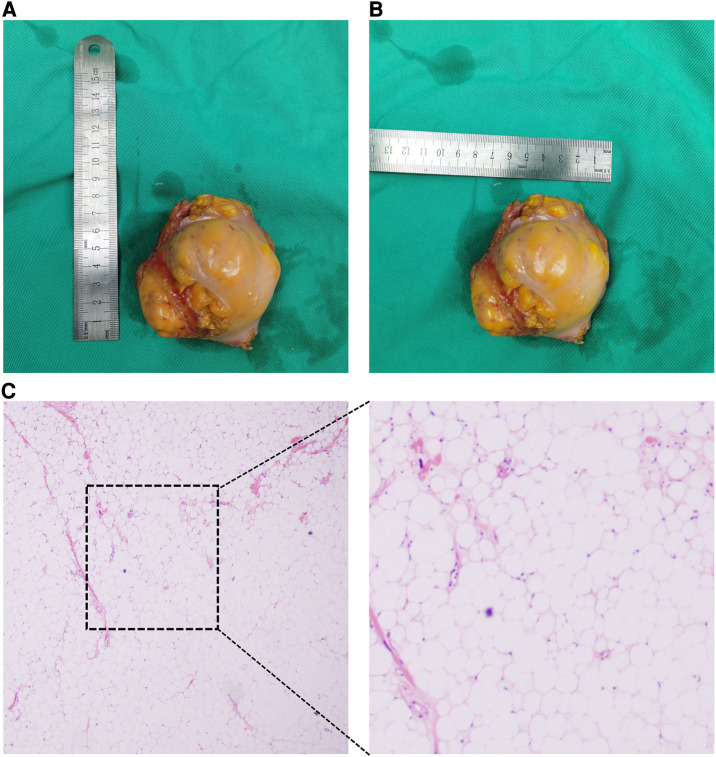
The macroscopical view and HE staining of resected mass. (**A**) Horizontal diameter of resected mass; (**B**) vertical diameter of resected mass; (**C**) HE staining result of resected mass.

**Figure 4:**
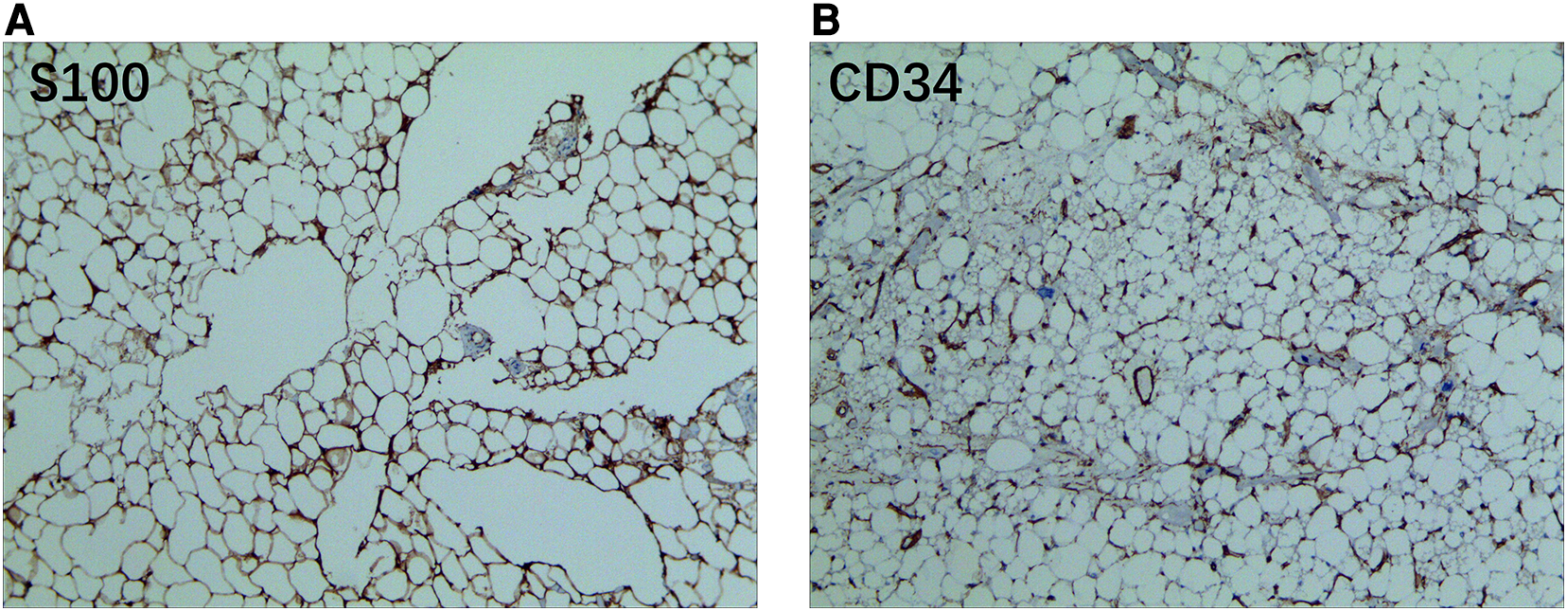
The immunohistochemical staining result of resected mass. (**A**) The mass exhibited a positive reaction to S100 staining; (**B**) the mass is positive to CD34 staining.

A 52-year-old female patient was admitted to the hospital due to recurring episodes of palpitations that were relieved by rest. Prior to this admission, the patient had visited a local hospital where echocardiography had suggested right atrial occupancy. Physical examination conducted after admission was non-specific. However, the electrocardiogram indicated frequent premature atrial pulses. Further evaluation by echocardiography revealed a 7.0 cm × 9.0 cm hypoechoic mass with limited motion in the right atrium, attached to the upper part of the interatrial septum with a wide base and partially protruding into the left atrium (Figure 1A). Mild tricuspid regurgitation was also noted. Computed tomography confirmed the presence of a massive intrapericardial mass occupying the interstitial space between the ascending aorta and the superior vena cava, which was poorly delineated from the right atrium (Figure 1B). The density of the mass was similar to that of subcutaneous fat, and a lipoma was suspected, although liposarcoma could not be ruled out. Due to the patient's symptomatic presentation and the potential for long-term complications, surgical removal of the mass was decided upon with the consent of the patient and family. During surgery, a large fatty tumor was discovered on the surface of the heart, almost entirely covering the right atrium and aortic root (Figure 2A). The mass was suspended for exposure of right atrium (Figure 2B). The right atrium was excised, exposing an intracardiac tumor that was attached to the atrial septum with a wide base, and infiltrated upward, penetrating the right atrial wall to form a large epicardial mass around the superior vena cava-right atrial junction and the aortic root (Figure 2C). As extensive resection was required, the defective atrium was patched using bovine pericardium (Figure 2D).The excised fatty mass was yellowish and soft, measuring approximately 7.9 cm × 8 cm (Figures 3A,B). Histopathological analysis of the specimen showed that the mass consisted predominantly of adipocytes (lipid droplets) with a moderate amount of fibrous tissue (Figure 3C). Immunohistochemical analysis revealed that the tumor was CK(−), MDM2(−), CDK-4(−), S-100(+), CD34(+), HNB45(−), Mela-A(−), 1% ki67(1%+), focal Desmin(+), and focal SMA(−), indicating a differentiated mature lipoma with mild fibrosis (Figure 4). Following surgery, the patient's palpitations subsided, and she made a rapid recovery, being discharged on the fifth postoperative day. At the six-month follow-up, there was no evidence of recurrence of the lipoma.

## Discussion

**Figure 5:**
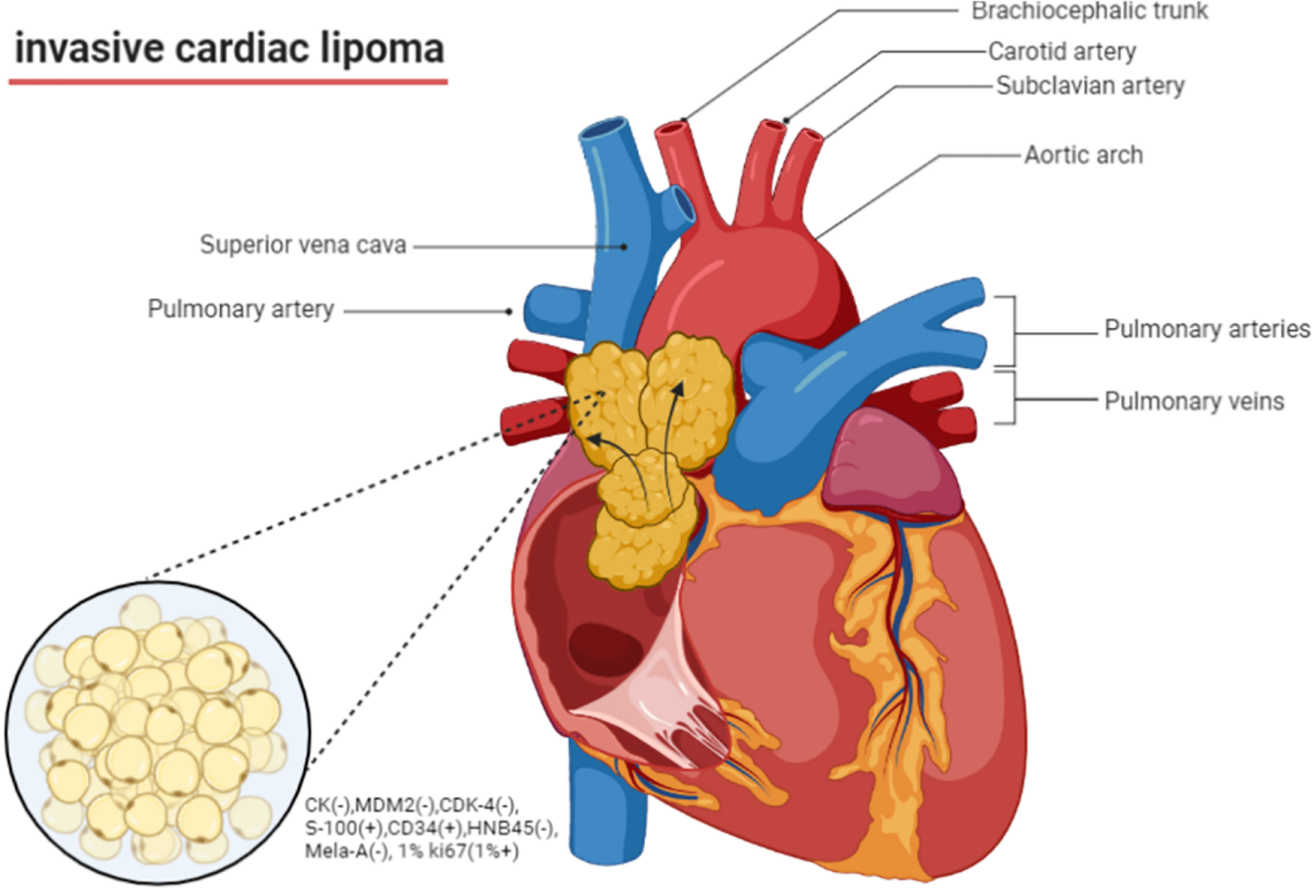
The schematic graph of invasive lipoma.

Cardiac lipomas are rare benign tumors that occur within the heart. They are composed of mature adipose tissue but are invasive like malignant tumor. This invasive lipoma originated from interatrial septum and across atrial wall form a giant lipoma covering super vena cava and root of aorta (Figure 5). Cardiac lipomas are rare tumors that account for 0.5% of primary cardiac tumors from 1976 to 1993 AFIP data (3). Invasive cardiac lipomas are even rarer, with only a few cases reported in the literatures (Table 1).

**Table 1:** The literatures of invasive cardiac Lipomas

Authors	Year	Age	Sex	Location	Shape	Size	Boundary	Mobility	Echogenicity
Present case	2022	52	F	RA	N/A	UCG: 7.0 cm × 9.0 cm	Well-demarcated	Mobile	N/A
Nepal (4)	2022	50	F	IVS	N/A	N/A	N/A	N/A	Hyperechoic
Fan (5)	2021	44	M	RA	N/A	Surgery: 4.0 cm × 5.0 cm, Surgery: 3.0 cm × 4.0 cm	N/A	Non-mobile	N/A
Bai (1)	2021	25	F	RA	Lobular	UCG: 4.2 cm × 4.6 cm × 4.3 cm	N/A	Mobile	Medial echo
Kadosaka (6)	2020	63	F	LV	N/A	CT: 3 cm	N/A	Mobile	N/A
Vriz (7)	2020	14	M	LV	N/A	/	N/A	N/A	N/A
Shah (8)	2019	61	F	RA	N/A	/	N/A	N/A	N/A
Naseerullah (9)	2018	67	F	RA	Oval	UCG: 4.0 × 5.7 cm	Smooth contours	Non-mobile	Solid
Kong (10)	2018	49	M	RA	N/A	UCG: 16.2 × 10.5 cm	N/A	N/A	Heterogeneous, hypoechoic
Kim (11)	2018	42	M	LV	Round	UCG: 3 × 3 cm	Well-circumscribed	N/A	Homogenous
D’Souza (12)	2017	33	M	RA, IAS	Irregular	UCG: 4.5 × 4.0 cm	N/A	Non-mobile	Heterogeneous
Saito (13)	2016	66	F	LV	Lobular	UCG: 5.4 × 3.5 cm	N/A	Mobile	Hyperechoic
Yuan (14)	2016	60	F	IAS	Round	UCG: 1.6 × 1.2 cm	N/A	N/A	Hyperechoic
Sakamoto (15)	2016	52	F	LV	Oval	UCG: 3.5 × 1.9 cm	N/A	Mobile	N/A
Zhang (16)	2016	49	F	RV	Oval	UCG: 6.1 × 3.1 cm	Ill-defined	N/A	Homogenous
Fang (17)	2016	48	F	RV, IVS	Irregular	UCG: 4.4 cm × 3.0 cm × 2.4 cm, UCG: 4.6 cm × 1.5 cm	N/A	Good mobility	Hyperechoic
Tanaka (18)	2015	77	F	LV	Oval	UCG: 2.5 cm × 2.8 cm	Well-demarcated	Movable	Hyperechoic
Barbuto (19)	2015	66	M	RA	Oval	UcG: 5.3 cm × 2.7 cm	N/A	N/A	Hypoechoic
Kilic (20)	2015	53	F	IAS	Oval	Surgery: 3.5 cm × 3 cm × 3 cm	N/A	Non-mobile	N/A
Zhu (21)	2015	48	F	Four cavities	Denticular	CT: 15 cm	N/A	N/A	Dense-echo
Wang (22)	2015	41	M	RV	Round	UCG: 4.0 cm × 2.5 cm	Regular, well-defined	N/A	Homogenous, hypoechoic
Girrbach (23)	2012	52	F	LA, LV	N/A	Surgery: 6.3 cm × 5.0 cm × 2.5 cm	N/A	N/A	Heterogeneous
Xie (17)	2012	48	F	RV	Oval	Surgery: 10 cm × 10 cm	N/A	N/A	Hyperechoic
Domoto (24)	2010	70	M	LV	Oval	UCG: 2.6 cm × 4.5 cm	N/A	Mobile	Hyperechoic
Joaquim (25)	2009	27	M	RA	Oval	UCG: 3.5 cm	N/A	N/A	Hyperechoic
Reddy (26)	2009	19	M	RV	Oval	UCG: 8.6 cm × 5 cm	N/A	N/A	Hyperechoic
Kitami (27)	2005	57	F	RV	N/A	Surgery: 5 cm	N/A	N/A	Echo-lucent
Agacdiken (28)	2005	18	F	RV, IVS	N/A	CT: 8 cm × 6 cm × 16 cm	N/A	N/A	Hyperechoic
Yoshitatsu (29)	2004	81	F	LV	Lobular	UCG: 1.5 cm × 1.5 cm	N/A	Fluttering	N/A
Courtis (30)	2004	79	F	RA, RV, IAS	N/A	/	N/A	N/A	Hyporrefringent
Schrepfer (31)	2003	31	F	RV	N/A	UCG: 4.5 cm–5 cm	No clear demarcation to the right ventricular myocardium	N/A	N/A
Chen (32)	2001	43	F	LV	N/A	Surgery: 10 cm × 8 cm × 3 cm	N/A	N/A	Hyperechoic
Bonamini (33)	2000	56	F	RV	N/A	/	N/A	N/A	Echo-free
Cooper (34)	1994	14	M	LA	Oval	MRl: 8.2 cm × 5 cm × 4.2 cm	Clear	N/A	N/A
King (35)	1993	17	M	RA	N/A	Surgery: 25 cm × 15 cm × 8 cm	N/A	N/A	Echo-free
Anderson (36)	1988	54	M	LV	N/A	UCG: 2 cm	N/A	Mobile	Hyperechoic
Zingas (37)	1983	20	F	IVS	Irregular	Surgery: 10 cm × 10 cm	N/A	Non-mobile	N/A
Harada (38)	1980	7	M	RV	Oval	/	N/A	N/A	Dense

The incidence of invasive cardiac lipomas is difficult to determine, as most cases are diagnosed incidentally during autopsy or surgery. The median age at diagnosis is around 50 years, and there is a slight female predominance (1, 17).

**Figure 6:**
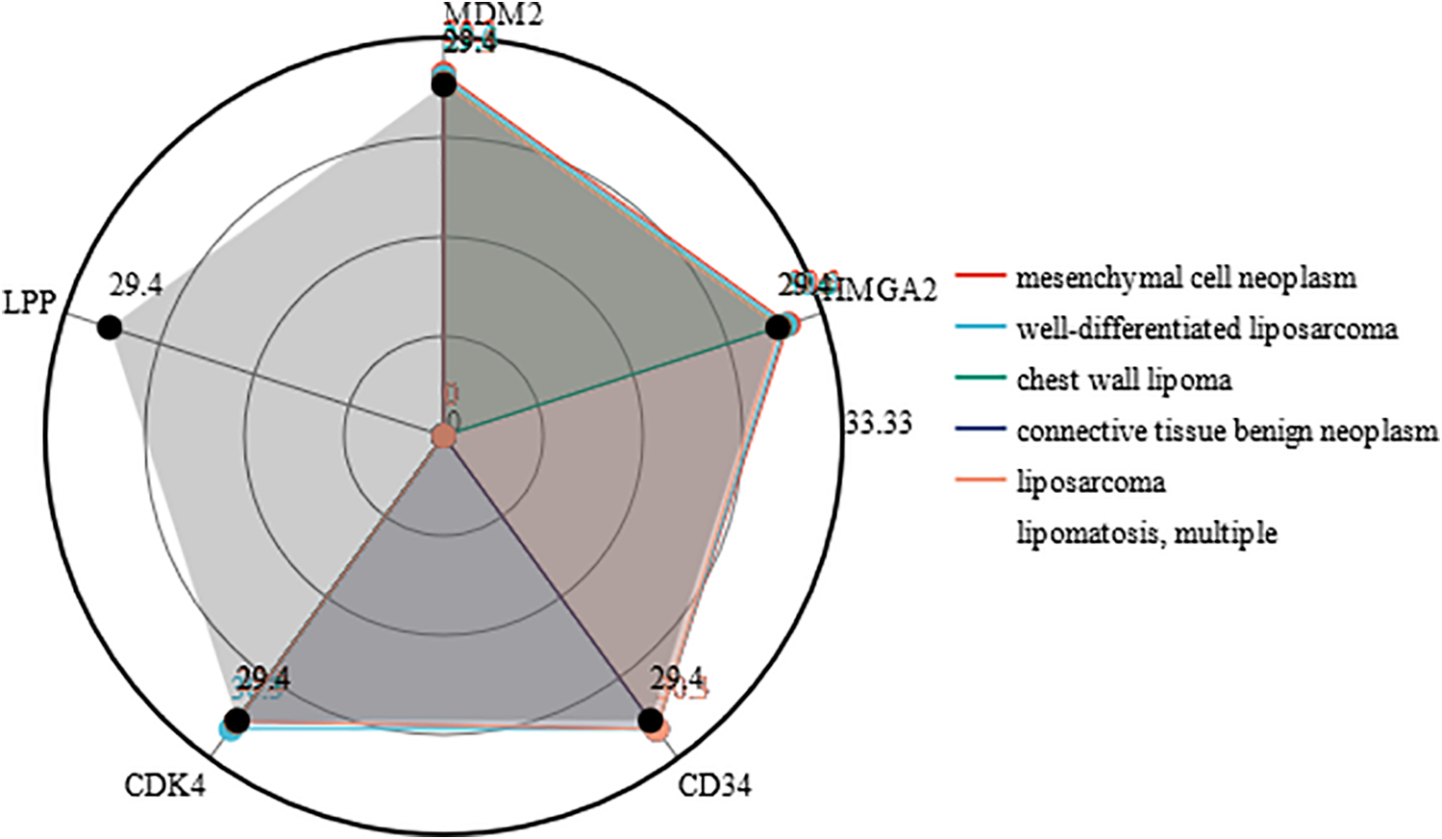
The disease enrichment of the related genes of invasive lipoma.

**Figure 7:**
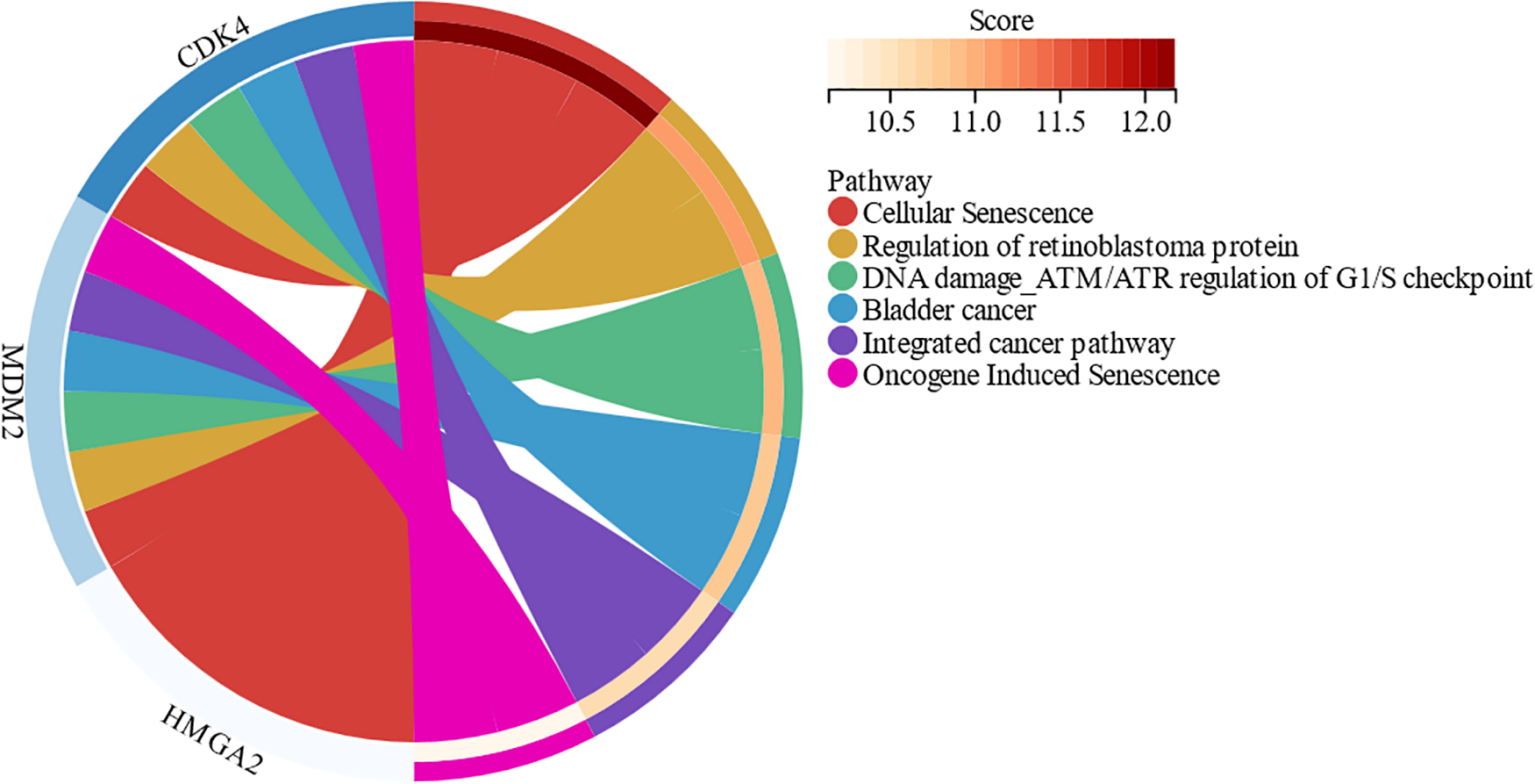
The signaling pathway enrichment of the related genes of invasive lipoma.

Invasive cardiac lipomas are characterized by their ability to invade the myocardium and infiltrate surrounding structures (6, 10, 15, 22). The mechanism of invasion is not well understood, but it is thought to be related to the ability of the tumor to secrete proteolytic enzymes that break down the extracellular matrix. Invasive cardiac lipomas may also be associated with a high degree of vascularity, which can facilitate tumor growth and invasion. Based on MalaCards database, invasive lipoma, or infiltrating lipoma, is related to mesenchymal cell neoplasm and well-differentiated liposarcoma. An important gene associated with invasive lipoma is MDM2 (MDM2 Proto-Oncogene) (Figure 6). However, the majority of intracardiac lipoma is negative for MDM2 amplification (12, 39). Among its related pathways are Cellular Senescence and Regulation of retinoblastoma protein (Figure 7).

The clinical presentation of invasive cardiac lipomas is variable and often nonspecific. Patients may present with symptoms such as chest pain, dyspnea, or palpitations, which can be attributed to the tumor's effect on cardiac function (7, 21, 31). Invasive cardiac lipomas can also cause arrhythmias, valvular dysfunction, or obstruction of blood flow, depending on their location within the heart (16). In some cases, the tumor may be asymptomatic and discovered incidentally during imaging studies (32).

The diagnosis of invasive cardiac lipomas is challenging, and a high degree of suspicion is required. The initial workup usually involves a comprehensive history and physical examination, including a detailed cardiac evaluation. Imaging studies such as echocardiography, cardiac magnetic resonance imaging (MRI), and computed tomography (CT) can provide valuable information about the size, location, and extent of the tumor (6, 24). In some cases, a biopsy may be necessary to confirm the diagnosis (1, 12, 17).

The management of invasive cardiac lipomas depends on several factors, including the size and location of the tumor, the patient's clinical presentation, and the degree of invasion. In general, small tumors that are asymptomatic and do not invade the myocardium may be monitored with periodic imaging studies. However, larger tumors or those that are causing significant symptoms or hemodynamic compromise may require surgical intervention. Surgical resection is the treatment of choice for invasive cardiac lipomas (11, 31). The surgical approach may vary depending on the location of the tumor and the degree of invasion. In some cases, a partial or complete excision of the tumor may be possible, while in others, the tumor may need to be debulked to relieve symptoms or prevent further invasion (25). In rare cases, a heart transplant may be necessary if the tumor is unresectable or has caused significant damage to the heart (40).

The prognosis for patients with invasive cardiac lipomas is generally good if the tumor is diagnosed and treated early. However, the prognosis may be poor if the tumor is large, invasive, or has caused significant damage to the heart (40). In some cases, the tumor may recur after surgical resection, and long-term surveillance is necessary (7, 40).

Generally speaking, invasive cardiac lipoma is an infrequent form of primary cardiac tumor, which can give rise to substantial morbidity and mortality. The accurate identification of invasive cardiac lipoma necessitates a heightened level of suspicion, along with comprehensive imaging investigations. Surgical intervention represents the preferred therapeutic approach for managing invasive cardiac lipoma; however, the intricacies associated with tumor localization and the potential for damage to adjacent structures can pose considerable surgical challenges. Consequently, a meticulous assessment of the potential risks and benefits associated with surgery becomes imperative in the management of patients diagnosed with invasive cardiac lipoma.

## References

[1] Bai R Zhang Y Wang H Yang J Sun D. Invasive cardiac lipoma diagnosis based on echocardiography: case report and literature review. J Clin Ultrasound. (2021) 49(4):408–12. 10.1002/jcu.22893 32748428

[2] Shu S Yuan H Kong X Wang J Wang J Zheng C. The value of multimodality imaging in diagnosis and treatment of cardiac lipoma. BMC Med Imaging. (2021) 21(1):71. 10.1186/s12880-021-00603-6 33858367 PMC8048252

[3] Amano J Nakayama J Yoshimura Y Ikeda U. Clinical classification of cardiovascular tumors and tumor-like lesions, and its incidences. Gen Thorac Cardiovasc Surg. (2013) 61(8):435–47. 10.1007/s11748-013-0214-8 23460447 PMC3732772

[4] Nepal S Deshmane SB Donovan K May A Chaudhuri D. Invasive lipoma of the interventricular septum, a rare benign cardiac mass with atypical presentation and management. J Investig Med High Impact Case Rep. (2022) 10:23247096221104469. 10.1177/23247096221104469 35726863 PMC9218626

[5] Fan W Liao B Li X. Lipoma across the wall of the right atrium. J Card Surg. (2021) 36(9):3390–2. 10.1111/jocs.15753 34157154

[6] Kadosaka T Tsujinaga S Iwano H Oyama-Manabe N Anzai T. Invasive cardiac lipoma complicating visceral inversion. JACC Case Rep. (2020) 2(10):1570–1. 10.1016/j.jaccas.2020.05.061 34317019 PMC8302107

[7] Vriz O Ahmed MM Kharabsheha S Aladmawi M Alamro B AlSomali A Ventricular tachycardia: beginning and ending fate of a benign invasive cardiac lipoma. Monaldi Arch Chest Dis. (2020) 90(3):1288. 10.4081/monaldi.2020.1288 32672428

[8] Shah OA Badran A Kaarne M Velissaris T. Right atrial and SVC infiltrating mass-the entity of infiltrating lipoma. J Cardiothorac Surg. (2019) 14(1):210. 10.1186/s13019-019-1015-7 31791367 PMC6889692

[9] Naseerullah FS Javaiya H Murthy A. Cardiac lipoma: an uncharacteristically large intra-atrial mass causing symptoms. Case Rep Cardiol. (2018) 2018:3531982. 10.1155/2018/3531982 29552360 PMC5818884

[10] Kong F Zhang W Guo Q. Multiple well-differentiated cardiac liposarcoma with a concomitant myocardial lipoma: a case report. Mol Clin Oncol. (2018) 9(6):617–21. 10.3892/mco.2018.1741 30546890 PMC6256261

[11] Kim YS Lee KH Choi SJ Baek WK. Cardiac lipoma arising from left ventricular papillary muscle: resect or not? J Thorac Cardiovasc Surg. (2018) 156(1):244–6. 10.1016/j.jtcvs.2018.01.040 29499869

[12] D'Souza J Shah R Abbass A Burt JR Goud A Dahagam C. Invasive cardiac lipoma: a case report and review of literature. BMC Cardiovasc Disord. (2017) 17(1):28. 10.1186/s12872-016-0465-2 28088193 PMC5237479

[13] Saito C Arai K Ashihara K Saito S Yamazaki K Multiple synchronous ventricular lipomas. J Echocardiogr. (2016) 14(2):85–6. 10.1007/s12574-016-0280-x 26898724

[14] Yuan SM. Postperfusion lung syndrome and related sequelae. J Thorac Dis. (2016) 8(5):E340–4. 10.21037/jtd.2016.03.44 27162696 PMC4842823

[15] Sakamoto H Tokunaga C Hiramatsu Y. Intramyocardial lipoma. J Card Surg. (2016) 31(11):689. 10.1111/jocs.12854 27677247

[16] Zhang HW Zhong MH Meng W Zhang EY Gu J Hu J Intramuscular lipoma as an unusual cause of right ventricular outflow tract obstruction. Echocardiography. (2016) 33(2):328–9. 10.1111/echo.13082 26494210

[17] Fang L He L Chen Y Xie M Wang J. Infiltrating lipoma of the right ventricle involving the interventricular septum and tricuspid valve: report of a rare case and literature review. Medicine (Baltimore). (2016) 95(3):e2561. 10.1097/MD.0000000000002561 26817909 PMC4998283

[18] Tanaka Y Yoshimuta T Yamagishi M Sakata K. Video-assisted transmitral resection of primary cardiac lipoma originated from the left ventricular apex. Eur Heart J Cardiovasc Imaging. (2015) 16(4):401. 10.1093/ehjci/jeu326 25588798

[19] Barbuto L Ponsiglione A Del Vecchio W Altiero M Rossi G De Rosa D Humongous right atrial lipoma: a correlative CT and MR case report. Quant Imaging Med Surg. (2015) 5(5):774–7. 10.3978/j.issn.2223-4292.2015.01.02 26682146 PMC4671973

[20] Kilic ID Alur I Alihanoglu YI Yildiz BS Bir F Ozcan AV. Lipoma in the right atrium. Herz. (2015) 40(1):150–2. 10.1007/s00059-013-3966-0 24154882

[21] Zhu J Liu Y Xi EP Zhu SB. A giant symptomatic cardiac lipoma recurring at the fifth year. Int J Clin Exp Med. (2015) 8(8):14173–5. 26550390 PMC4613075

[22] Wang Y Ma C Yang J Gu T. Incomplete excision or postoperative hematoma: primary right ventricular intramyocardial lipoma involving the right ventricular outflow tract. J Med Ultrason (2001). (2015) 42(4):541–5. 10.1007/s10396-015-0632-6 26576979

[23] Girrbach F Mohr FW Misfeld M. Epicardial lipoma–a rare differential diagnosis in cardiovascular medicine. Eur J Cardiothorac Surg. (2012) 41(3):699–701. 10.1093/ejcts/ezr020 22345190

[24] Domoto S Nakano K Kodera K Sasaki A Asano R Ikeda M Cardiac lipoma originating from the left ventricular apex diagnosed using the magnetic resonance imaging fat suppression technique: report of a case. Surg Today. (2010) 40(9):871–3. 10.1007/s00595-009-4146-y 20740352

[25] Joaquim MR Braile DM Arruda MVFD Soares MJF. Right atrial lipoma resection and partial reconstruction using bovine pericardium. Rev Bras Cir Cardiovasc. (2009) 24(2):239–41. 10.1590/S0102-76382009000200022 19768305

[26] Reddy VK Faulkner M Bandarupalli N Nanda NC Singh P Dutta R Incremental value of live/real time three-dimensional transthoracic echocardiography in the assessment of right ventricular masses. Echocardiography. (2009) 26(5):598–609. 10.1111/j.1540-8175.2009.00952.x 19438700

[27] Kitami A Suzuki S Suzuki T. Giant intrapericardial extracavitary lipoma: report of a case. Surg Today. (2005) 35(9):789–91. 10.1007/s00595-005-3002-y 16133678

[28] Agacdiken A Gurbuz Y Ciftci E Omay O Vural A Ural D. Cardiac lipoma in a patient with proven arrhythmogenic right ventricular dysplasia: a case report. A huge intramyocardial lipoma. Int J Cardiovasc Imaging. (2005) 21(4):463–7. 10.1007/s10554-004-7024-0 16047131

[29] Yoshitatsu M Nomura F Katayama A Tamura K Katayama K Ihara K. Intraoperative view of cardiac lipoma by using a compact camera. Asian Cardiovasc Thorac Ann. (2004) 12(1):89. 10.1177/021849230401200123 14977753

[30] Courtis J Marani L Amuchastegui LM Rodeiro J. Cardiac lipoma: a rare cause of right-to-left interatrial shunt with normal pulmonary artery pressure. J Am Soc Echocardiogr. (2004) 17(12):1311–4. 10.1016/j.echo.2004.06.031 15562273

[31] Schrepfer S Deuse T Detter C Treede H Koops A Boehm DH Successful resection of a symptomatic right ventricular lipoma. Ann Thorac Surg. (2003) 76(4):1305–7. 10.1016/S0003-4975(03)00523-X 14530040

[32] Chen HM Chiu CC Lee CS Lai WD Lin YT. Intractable ventricular tachycardia in a patient with left ventricular epicardial lipoma. J Formos Med Assoc. (2001) 100(5):339–42. 11432314

[33] Bonamini R Rosettani E Mangiardi L Pinneri F Cirillo S. A large false aneurysm of the right ventricle within a giant epicardial lipoma. Chest. (2000) 117(2):601–3. 10.1378/chest.117.2.601 10669714

[34] Cooper MJ deLorimier AA Higgins CB Van Hare GF Enderlein MA. Atrial flutter-fibrillation resulting from left atrial compression by an intrapericardial lipoma. Am Heart J. (1994) 127(4 Pt 1):950–1. 10.1016/0002-8703(94)90573-8 8154443

[35] King SJ Smallhorn JF Burrows PE. Epicardial lipoma: imaging findings. AJR Am J Roentgenol. (1993) 160(2):261–2. 10.2214/ajr.160.2.8424329 8424329

[36] Anderson DR Gray MR. Mitral incompetence associated with lipoma infiltrating the mitral valve. Br Heart J. (1988) 60(2):169–71. 10.1136/hrt.60.2.169 3415877 PMC1216542

[37] Zingas AP Carrera JD Murray III CA Kling GA. Lipoma of the myocardium. J Comput Assist Tomogr. (1983) 7(6):1098–100. 10.1097/00004728-198312000-00034 6630645

[38] Harada K Seki I Kobayashi H Okuni M Sakurai I. Lipoma of the heart in a child. Clinical, echocardiographic, angiographic, and pathological features. Jpn Heart J. (1980) 21(6):903–10. 10.1536/ihj.21.903 7463728

[39] Bois MC Bois JP Anavekar NS Oliveira AM Maleszewski JJ. Benign lipomatous masses of the heart: a comprehensive series of 47 cases with cytogenetic evaluation. Hum Pathol. (2014) 45(9):1859–65. 10.1016/j.humpath.2014.05.003 24996689

[40] Peters CM Kalra N Sorrell VL. Extensive recurrent cardiac lipoma. J Cardiovasc Comput Tomogr. (2009) 3(4):282–3. 10.1016/j.jcct.2009.03.007 19577219

